# Aronia berry extract inhibits TNF-α-induced vascular endothelial inflammation through the regulation of STAT3

**DOI:** 10.29219/fnr.v63.3361

**Published:** 2019-08-16

**Authors:** Tomomi Iwashima, Yuki Kudome, Yoshimi Kishimoto, Emi Saita, Miori Tanaka, Chie Taguchi, Satoshi Hirakawa, Nobu Mitani, Kazuo Kondo, Kaoruko Iida

**Affiliations:** 1 Department of Food and Nutritional Sciences, Graduate School of Humanities and Sciences, Ochanomizu University, Tokyo, Japan; 2 Endowed Research Department “Food for Health,” Ochanomizu University, Tokyo, Japan; 3 Pola Chemical Industries Inc., Kanagawa, Japan; 4 Institute of Life Innovation Studies, Toyo University, Gunma, Japan; 5 Institute for Human Life Innovation, Ochanomizu University, Tokyo, Japan

**Keywords:** aronia berry, polyphenol, atherosclerosis, STAT3, vascular endothelial cell, inflammation

## Abstract

**Background:**

Inflammation in endothelial cells induces production of inflammatory cytokines and monocytes adhesion, which are crucial events in the initiation of atherosclerosis. Aronia berry (*Aronia meranocalpa*), also called black chokeberry, contains abundant anthocyanins that have received considerable interest for their possible relations to vascular health.

**Objective:**

The aim of this study was to investigate whether an anthocyanin-rich extract obtained from aronia berry can attenuate inflammatory responses in vascular endothelial cells.

**Methods:**

As a model of vascular endothelial inflammation, human umbilical vein endothelial cells (HUVECs) pretreated with aronia berry extract were stimulated with tumor necrosis factor-alpha (TNF-α). The expression levels of cytokines and adhesion molecules were analyzed. To investigate the effects of aronia berry extract on the adhesion of THP-1 monocytic cell, the static adhesion assay was carried out. The possible molecular mechanisms by which aronia berry extract regulated vascular inflammatory responses were explored.

**Results:**

The mRNA expressions of interleukins (IL-1β, IL-6, and IL-8) and monocyte chemoattractant protein-1 (MCP-1) upregulated by TNF-α were significantly suppressed by pretreatment with aronia berry extract. Aronia berry extract decreased TNF-α-induced monocyte/endothelial adhesion and suppressed vascular cell adhesion molecule-1 (VCAM-1) expression, but did not affect intercellular adhesion molecule-1 (ICAM-1) expression. Moreover, aronia berry extract decreased the phosphorylation of signal transducer and activator of transcription 3 (STAT3) and the nuclear levels of STAT3 and interferon regulatory transcription factor-1 (IRF1). The nuclear translocation of nuclear factor-kappa B (NF-κB) was not inhibited by aronia berry extract.

**Conclusion:**

Aronia berry extract could exert anti-atherosclerotic effects on TNF-α-induced inflammation through inhibition of STAT3/IRF1 pathway in vascular endothelial cells.

## Popular scientific summary

- Aronia berry contains abundant anthocyanins and recently its functionality attracts attention.
- The effect of aronia berry extract on vascular endothelial inflammation was investigated in the cultured human endothelial cells stimulated with TNF-α.
- Aronia berry extract attenuated the expressions of inflammatory cytokines and cell adhesion molecules through the inhibition of STAT3/IRF1 pathway.
- Anti-inflammatory effect of aronia berry extract may be useful to reduce risk factors for atherosclerosis.

Vascular endothelial inflammation is well known to be an initial step in atherosclerosis (1, 2). Vascular cells regulate the inflammatory process through the expression of cytokines (e.g. tumor necrosis factor-alpha [TNF-α], interleukin [IL]-1β, IL-6), chemokines, growth factors, and adhesion molecules. When vascular endothelial cells undergo inflammatory activation, the activated endothelial cells further increase their expression of chemokines/chemoattractants such as IL-8 (also known as CXCL8) and monocyte chemoattractant protein-1 (MCP-1) which are involved in attracting leukocytes, and cell adhesion molecules (CAMs) such as vascular cell adhesion molecule-1 (VCAM-1) and intercellular adhesion molecule-1 (ICAM-1). This promotes the adherence of the inflammatory cells, including monocytes, neutrophils, lymphocytes, and macrophages and the induction of additional cytokines. Adherent inflammatory cells invade under the endothelium to form atheromatous plaques, and their effector molecules promote the progression of plaques (3, 4). Thus, medications or nutraceuticals that suppress the expression of inflammatory cytokines/chemokines and adhesion molecules are promising candidates for the treatment and prevention of atherosclerotic diseases.

Regarding the mechanisms underlying the regulation of the expression of inflammatory cytokines and adhesion molecules, the involvement of the nuclear factor-kappa B (NF-κB) pathway and the Janus kinase (JAK)/signal transducer and activator of transcription (STAT) pathway is well known (5). These pathways are activated by stimulation such as that by TNF-α, IL-1β, or IL-6. The activation of NF-κB signaling has been established at different stages of atherosclerosis, beginning from plaque formation and on to the destabilization and rupture of plaques. The NF-κB pathway also mediates angiogenic, apoptotic, and neoplastic processes (6). It was reported that the abnormal activation of STAT and interferon regulatory transcription factor (IRF) signaling pathways is involved in various human diseases (including cardiovascular diseases), consequently attracting attention as highly interesting therapeutic targets (7, 8). IRFs are a family of transcription factors that play important roles in inflammatory regulation, antiviral responses, cytokine signaling, and cell death, growth, and differentiation (9). The JAK/STAT3 pathway is upstream in the regulation of IRF1 (10). Notably, it was reported that the inhibition of the JAK/STAT pathway reduced atherogenesis (11), and that STAT3 tyrosine phosphorylation is critical for IL-1β and IL-6 production in response to inflammatory stimuli (12).

Epidemiological, clinical, and basic studies indicate that anthocyanins, one class of flavonoids widely available in berries, exert protective effects against atherosclerotic diseases by acting on multiple targets in the vascular system (13, 14). Aronia berry (*Aronia meranocalpa*), known as black chokeberry, is a shrub native to North America and is commonly consumed as juice, wine, and jam (15). Aronia berry contains abundant polyphenols, mainly anthocyanins, and also quercetin and chlorogenic acid (16). Aronia berry is reported to contain three times as much anthocyanins as blueberry (17). The functionality of aronia berry has attracted attention; antioxidative, anti-inflammatory, antimutagenic, antidiabetic, and hepatoprotective activities have been demonstrated (15, 18). It was reported that aronia berry extract showed a greater effect on endothelium-dependent relaxation in porcine coronary arteries compared with extracts of other berries (19). Moreover, aronia berry extract has been shown to reduce blood cardiovascular risk markers such as oxidized-low-density lipoproteins (LDL), C-reactive protein (CRP), IL-6, soluble-ICAM-1, soluble-VCAM-1, and MCP-1 in patients after myocardial infarction (20). However, the mechanisms by which aronia berry protects against vascular inflammation remain to be fully elucidate.

In the present study, we explored the effects of aronia berry extract on inflammation in vascular endothelial cells, which are closely involved in the progression of arteriosclerosis. We conducted *in vitro* experiments using human umbilical vein endothelial cells (HUVECs) under stimulation with TNF-α as a model of vascular endothelial inflammation.

## Materials and methods

### Materials

A concentrated ethanol extract of aronia berry was provided by POLA Chemical Industries Inc. (Kanagawa, Japan). According to a high-performance liquid chromatography (HPLC) analysis conducted by the manufacturer, the total anthocyanins content of the aronia berry extract powder was 36.49%, which consisted of 23.52% cyanidin-3-O-galactoside, 10.48% cyanidin-3-O-arabinoside, 1.36% cyanidin-3-O-xyloside, and 1.13% cyanidin-3-O-glucoside. The powder extract was dissolved in deionized water at 5 mg/mL and used in the experiments. RPMI-1640 medium, Hank’s balanced salts solution (HBSS), and 2’,7’-bis(2-carboxyethyl)-5(6)-carboxyfluorescein (BCECF) were purchased from Sigma-Aldrich (St. Louis, MO, USA). Fetal bovine serum (FBS), penicillin/streptomycin, novoheparin, and recombinant human fibroblast growth factor (hFGF) basic were obtained from Biowest (Nuaillé, France), GIBCO (Grand Island, NY), Mochida Pharmaceutical (Tokyo), and R&D Systems (Minneapolis, MN), respectively.

### Cell culture and treatment

HUVECs were obtained from Lonza (Basel, Switzerland), grown in RPMI-1640 medium supplemented with 20% FBS, 1% penicillin/streptomycin, 0.5% novoheparin, and 0.05% recombinant hFGF basic at 37°C and 5% CO_2_. HUVECs were pretreated with aronia berry extract (0–25 μg/mL) and then stimulated with TNF-α (10 ng/mL). The concentration of aronia berry extract added to the cells was set within the range of noncytotoxic based on the results of MTT assay (data not shown).

### Real-time RT-PCR analysis

RNA was extracted from cells with the total RNA extract reagent RNAiso Plus (Takara Bio, Shiga, Japan). We synthesized complementary DNAs from 2 μg of the total RNA by using a High Capacity cDNA Reverse Transcription Kit (Thermo Fisher Scientific, Rockford, IL). A real-time polymerase chain reaction (PCR) was performed on an ABI 7300 cycler (Applied Biosystems, Foster City, CA) with Power SYBR Green PCR mix (Thermo Fisher Scientific). We used r18s rRNA as the endogenous control. For all samples, the quantity was determined from the relative standard curve and normalized with an endogenous control. The primer sequences are as follows: for r18s, forward 5’-ACT CAA CAC GGG AAA CCT CAC-3’ and reverse 5’-CAG ACA AAT CGC TCC ACC AA-3’; for IL-1β (*IL1B*), forward 5’-CTG TAC GAT CAC TGA ACT GC-3’ and reverse 5’-CAC CAC TTG TTG CTC CAT ATC-3’; for IL-6 (*IL6*), forward 5’-GGT ACA TCC TCG ACG GCA TC-3’ and reverse 5’-GCC TCT TTG CTG CTT TCA CAC-3’; for gp130 (*IL6ST*), forward 5’-CTG GGA GTG CTG TTC TGC TTT-3’ and reverse 5’-GCC TTG GAG GAG TGT GAG GT-3’; for IL-8 (*CXCL8*), forward 5’-AAA CTG GGT GCA GAG GGT TG-3’ and reverse 5’-TGG CAT CTT CAC TGA TTC TTG G-3’; for MCP-1 (*CCL2*), forward 5’-ATC ACC AGC AGC AAG TGT CC-3’ and reverse 5’-CAA GTC TTC GGA GTT TGG GTT T-3’; for VCAM-1 (*VCAM1*), forward 5’-CAT GGA ATT CGA ACC CAA ACA-3’ and reverse 5’-GAC CAA GAC GGT TGT ATC TCT GG-3’; and for ICAM-1 (*ICAM1*), forward 5’-GGG CAG TCA ACA GCT AAA ACC TT-3’ and reverse 5’-CAC CTG GCA GCG TAG GGT AA-3’.

### Western blot analysis

We isolated total cell and nuclear extract lysates by using a protein extraction reagent and NE-PER Nuclear and Cytoplasmic Extraction Reagents (Thermo Fisher Scientific) containing protease inhibitor cocktail (NacalaiTesque, Kyoto, Japan). Aliquots of protein were separated bysodium dodecyl sulfate-polyacrylamide gel electrophoresis (SDS-PAGE) (10%) and transferred to an Immobilon-P membrane (Millipore, Bedford, MA). After blocking, the membranes were incubated with primary antibodies: anti-gp130, anti-NF-κB, anti-VCAM-1, anti-ICAM-1, anti-LaminB (Santa Cruz Biotechnology, Santa Cruz, CA), anti-stat3, anti-phospho-stat3, anti-IRF-1, anti-β-actin, and anti-GAPDH (Cell Signaling Technology, Danvers, MA). Each bound antibody was then detected with a horseradish peroxidase-conjugated anti-rabbit or anti-mouse IgG secondary antibody. Chemiluminescent detection of specific proteins was developed with the use of ECL Select Western Blotting Detection Reagent (GE Healthcare, Little Chalfont, UK). All signals were captured by the LAS-4000 system (Fujifilm, Tokyo).

### Adhesion assay

We obtained the human monocytic cell line THP-1 from the RIKEN Cell Bank (Ibaraki, Japan). THP-1 cells were incubated with the fluorescent dye BCECF for 20 min at 37°C. The fluorescently labeled THP-1 cells were added to the HUVECs and incubated for10 min. The unbound THP-1 cells were washed away, and fluorescent images were obtained with a fluorescence microscope. The fluorescent intensity of the adherent THP-1 cells was also measured at Ex.482 nm/Em.528 nm by a PowerScan-HT (BioTek Instruments, Tokyo).

### Statistical analysis

Values are presented as the mean ± SD. We performed a one-way analysis of variance (ANOVA) followed by Tukey’s post hoc test to compare treatment groups. *P* values < 0.05 were accepted as significant. All results were analyzed with the GraphPad Prism 5 software package (GraphPad Software, La Jolla, CA).

## Results

### Aronia berry extract reduced the expression of inflammatory cytokines and chemokines

**Fig. 1:**
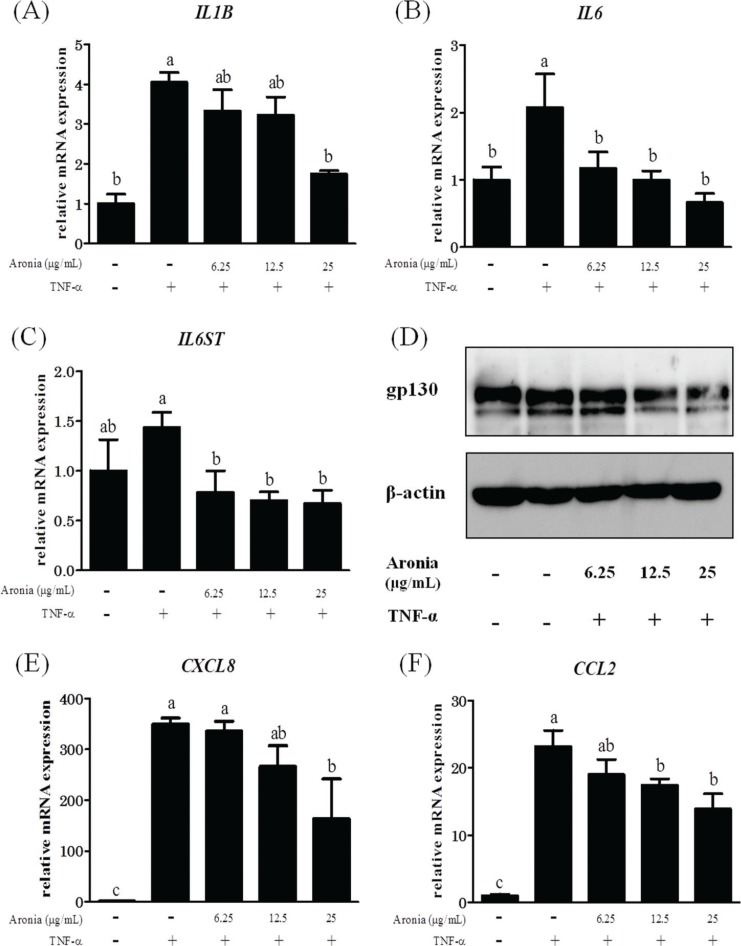
Effects of aronia berry extract on inflammation-related factors. HUVECs were pretreated with extract (0–25 μg/mL) for 24 h and then stimulated with or without TNF-α (10 ng/mL) for 3 h. The mRNA expressions for *IL1B* (A), *IL6* (B), *IL6ST* (gp130) (C), *CXCL8* (IL-8) (E), and *CCL2* (MCP-1) (F) were measured by real-time RT-PCR. Values are mean ± SD (*n* = 3). Different letters indicate a significant difference between groups (Tukey’s test after one-way ANOVA, *p* < 0.05). (D) The gp130 protein expression was analyzed by western blotting. Representative blots are shown.

As mentioned above, IL-1β, IL-6, IL-8, and MCP-1 are important factors in the development of atherosclerosis. As shown in Fig. 1A, B, the enhanced expression of mRNA for *IL1B* and *IL6* by the addition of TNF-α was significantly suppressed by aronia berry extract. Moreover, *IL6ST* (gp130), a signaling molecule of IL-6, was suppressed by aronia berry extract at both the mRNA and protein levels (Fig. 1C, D). Aronia berry extract also suppressed the increases in the mRNA expressions of *CXCL8* (IL-8) and *CCL2* (MCP-1) (Fig. 1E, F).

### Aronia berry extract decreased the expression of VCAM-1 but not ICAM-1

**Fig. 2:**
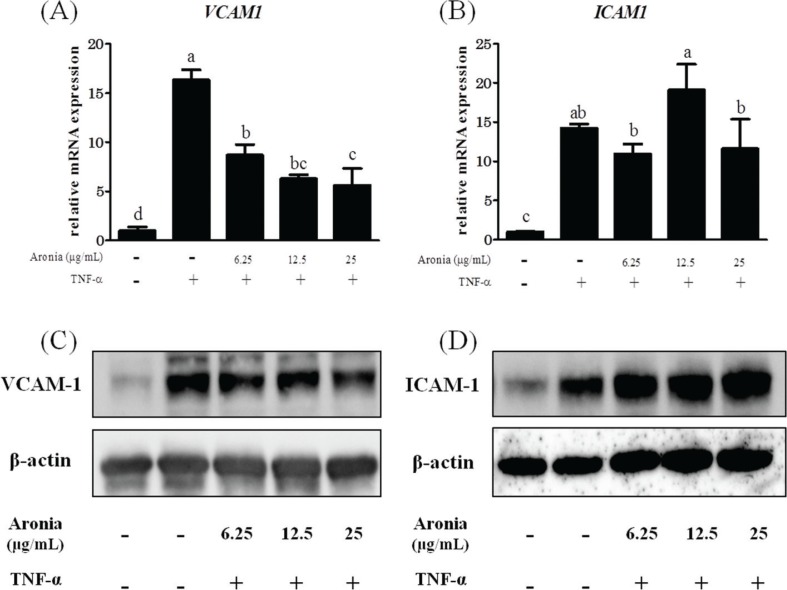
Effects of aronia berry extract on cell adhesion molecules. HUVECs were pretreated with aronia berry extract (0–25 μg/mL) for 24 h and then stimulated with TNF-α (10 ng/mL) or not stimulated for 3 h. The mRNA expression for *VCAM1* (A) and *ICAM1* (B) was measured by real-time RT-PCR. Values are mean ± SD (*n* = 3). Different letters indicate a significant difference between groups (Tukey’s test after one-way ANOVA, *p* < 0.05). (C, D) The protein expression was analyzed by western blotting. Representative blots are shown.

CAMs are greatly involved in plaque formation by mediating leukocyte migration. Here we observed that aronia berry extract concentration-dependently reduced TNF-α-stimulated VCAM-1 induction at both the mRNA and protein levels (Fig. 2A, C). However, the expression of ICAM-1 was unchanged (Fig. 2B, D).

### Aronia berry extract inhibited THP-1 cells’ adhesion to HUVECs

**Fig. 3:**
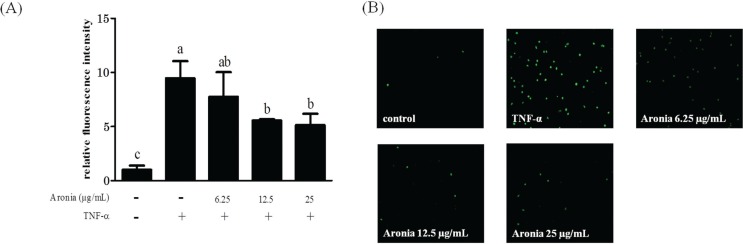
Effect of aronia berry extract on THP-1 cells’ adhesion to HUVECs. HUVECs were pretreated with aronia berry extract (0–25 μg/mL) for 24 h and then stimulated with TNF-α (10 ng/mL) or not stimulated for 3 h. The fluorescence-labeled THP-1 cells were added to the HUVECs and allowed to adhere for 10 min. (A) The fluorescent intensity of the adherent THP-1 cells was measured using BCECF dye (excitation 485 nm/emission 528 nm). Values are means ± SD (*n* = 3). Different letters indicate a significant difference between groups (Tukey’s test after one-way ANOVA, *p* <0.05). (B) The fluorescent images were obtained using a fluorescence microscope.

To investigate the effect of aronia berry extract on monocyte-endothelial interactions, we performed an adhesion assay under static (no shear stress) conditions. The stimulation with TNF-α significantly increased the adhesion of THP-1 cells to HUVECs. In contrast, pretreatment with aronia berry extract (≥12.5 μg/mL) significantly reduced the adherent cells to HUVECs (Fig. 3A, B).

### Aronia berry extract downregulated STAT3/IRF1, but not NF-κB

**Fig. 4:**
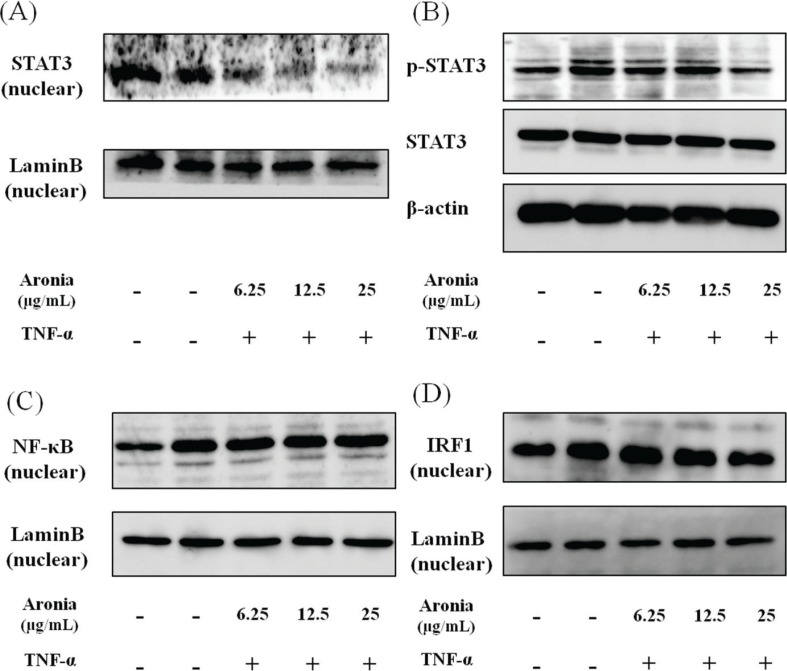
Effects of aronia berry extract on STAT3, NF-κB, and IRF1. HUVECs were pretreated with aronia berry extract (0–25 μg/mL) for 24 h and then stimulated with TNF-α (10 ng/mL) or not stimulated for 15 min (for B), 30 min (for D), 1 h (for A) or 2 h (for C). The nuclear levels of STAT3 (A), NF-κB (C), and IRF1 (D), and the phosphorylation of STAT3 (B) were analyzed by western blotting. Representative blots are shown.

Inflammatory molecules are known to be regulated by the nuclear transcription factors NF-κB and STAT3. To investigate the mechanism underlying the anti-inflammatory effects of aronia berry extract, we examined the nuclear translocation of NF-κB and STAT3. The aronia berry extract inhibited the TNF-α-induced STAT3 phosphorylation and suppressed the nuclear expression (Fig. 4A, B), but it did not affect the nuclear expression of NF-κB (Fig. 4C). Moreover, the TNF-α-induced nuclear translocation of IRF1, a transcription regulatory factor downstream of STAT3, was reduced by aronia berry extract (Fig. 4D).

## Discussion

The results of our present experiments revealed that aronia berry extract attenuated TNF-α-induced proinflammatory changes in endothelial cells, which are involved in the upregulation of inflammatory cytokines and chemokines (IL-1β, IL-6, IL-8, and MCP-1) and an adhesion molecule (VCAM-1) and an increase in endothelial-monocyte adhesion. Aronia berry extract also decreased the expression of gp130, a signaling molecule of IL-6. Interestingly, aronia berry extract downregulated the TNF-α-induced VCAM-1 expression but not ICAM-1 expression, and it decreased the nuclear expressions of STAT3 and IRF1. Our data indicate that the anti-inflammatory effects of aronia berry extract are exerted, at least in part, through a suppression of STAT3 activation.

An overexpression of inflammatory cytokines/chemokines, such as IL-1β, -6, -8, and MCP-1, induces cell migration and adhesion, angiogenesis, and vascular permeability to exacerbate atherosclerosis (21–25). In the present study, the pretreatment with aronia berry extract significantly decreased the mRNA expressions of IL-1β, -6, -8, and MCP-1 in TNF-α-stimulated HUVECs. It is known that IL-6 production is enhanced by IL-1 in vascular endothelial cells (26). Sawada et al. (27) reported that a synergistic effect of IL-1β and IL-6 may regulate the progression of inflammation through the induction of IL-6 signal transducer gp130 expression. We also observed herein that aronia berry extract downregulated the TNF-α-induced VCAM-1 protein and mRNA expression in a concentration-dependent manner, whereas it had no effect on ICAM-1 expression. The effect of aronia berry extract on the expression of adhesion molecules was confirmed at the functional level by our THP-1/HUVEC adhesion assay, which demonstrated that aronia berry extract reduces the monocyte adhesion induced by TNF-α.

Aronia berry extract contains a rich amount of anthocyanins, mainly cyanidin-3-O-galactoside and cyanidin-3-O-arabinoside, which we suspect contributed to our present findings. Earlier studies have demonstrated that several anthocyanins or anthocyanin-rich plant extracts inhibited the expression of inflammatory cytokines/chemokines and adhesion molecules and the adhesion of monocytes to vascular endothelial cells (28–31). As we noted earlier, regarding the mechanisms underlying the regulation of these inflammatory mediators’ expression, the involvement of the NF-κB pathway and JAK/STAT pathway are well established (5). Speciale et al. reported that cyanidin-3-O-glucoside downregulated the TNF-α-stimulated NF-κB signal transduction pathway, which responds to oxidative signals, and thus inhibited both VCAM-1 and ICAM-1 induction in HUVECs (32). Luo et al. reported that cyanidin-3-O-glucoside diminished the activation of STAT3 (33). In contrast to Speciale et al.’s study, our present results showed that aronia berry extract downregulated TNF-α-induced VCAM-1 but not ICAM-1 expression and that the NF-κB pathway was not affected. Similar results were shown in studies using Edelweiss extract or proanthocyanidins from grape seeds (34, 35). VCAM-1 and ICAM-1 have similar structures (36), but they are regulated by different mechanisms and show different functions at the initiation of lesions of atherosclerosis (37). Although some expression of ICAM-1 protein and mRNA is found in unstimulated cells, VCAM-1 expression is absent in unstimulated cells but is rapidly induced by cytokines. It has been reported that the VCAM-1 promoter region contains several transcription factor-binding motifs, including NF-κB, activator protein-1 (AP-1), IRF1, and transcription factor genes binding to DNA sequence GATA (GATAs) (38–43). On the contrary, the IRF1 and GATAs binding motifs are not present in the ICAM-1 promoter.

Our present results showed that aronia berry extract had no effect on the NF-κB pathway, but it suppressed the STAT3/IRF1 pathway through an inhibition of the phosphorylation of STAT3. As a result, the expression of inflammatory cytokines such as IL-1β and IL-6 decreased, and the expression of VCAM-1 (which is regulated by IRF1) was suppressed by aronia berry extract. The aronia berry extract used in this study at 25 μg/mL contained approximately 20 μmol/L of cyanidin glycosides. Similar concentrations were used in the studies of anthocyanins (1–100 μmol/L) (28–30, 32, 33). Taken together, the anthocyanin-rich aronia berry extract may attenuate the expressions of inflammatory cytokines and cell adhesion molecules through the inhibition of the activation of STAT3 in TNF-α-stimulated HUVECs. Recently, Warner et al. investigated the activity of physiologically relevant anthocyanin metabolite signatures, derived from a previous pharmacokinetics study of 500 mg ^13^C-labeledcyanidin-3-glucoside, on TNF-α-induced VCAM-1 and IL-6 production. They found that the greatest inhibition of VCAM-1 was observed in response to the 24 h metabolite signature, suggesting the metabolites of lower intestinal microbial origin are responsible for anti-inflammatory effects (44). It should be important to investigate whether metabolites of aronia berry extract will have differential biological activities to their precursor structures and that metabolites in combination may have additive or synergistic effects.

In conclusion, we observed that aronia berry extract could regulate the expressions of inflammatory cytokines and cell adhesion molecules through the inhibition of STAT3 activation in TNF-α-induced vascular endothelial cells. Our results indicate that aronia berry extract may be effective in the reduction of risk factors associated with atherosclerosis, but further investigations of the detailed molecular mechanisms and the *in vivo* efficacy of aronia berry extract are needed.

## Conflict of interest and funding

This work was partly supported by JSPS KAKENHI (Grant nos. 15H02895, 16H03033, and 17K00847) from the Japan Society for the Promotion of Science. The authors declare no potential conflicts of interest.

## References

[1] Libby P, Ridker PM, Maseri A Inflammation and atherosclerosis. Circulation 2002; 105: 1135–43. doi: 10.1161/hc0902.104353. 11877368

[2] Libby P Inflammation in atherosclerosis. Arterioscler Thromb Vasc Biol 2012; 32: 2045–51. doi: 10.1161/atvbaha.108.179705. 22895665 PMC3422754

[3] Blankenberg S, Barbaux S, Tiret L Adhesion molecules and atherosclerosis. Atherosclerosis 2003; 170: 191–203. doi: 10.1016/S0021-9150(03)00097-2. 14612198

[4] Manduteanu I, Simionescu M Inflammation in atherosclerosis: a cause or a result of vascular disorders? J Cell Mol Med 2012; 16: 1978–90. doi: 10.1111/j.1582-4934.2012.01552.x. 22348535 PMC3822968

[5] Tedgui A, Mallat Z: Cytokines in atherosclerosis: pathogenic and regulatory pathways. Physiol Rev 2006; 86: 515–81. doi: 10.1152/physrev.00024.2005. 16601268

[6] Pamukcu B, Lip GY, Shantsila E The nuclear factor – kappa B pathway in atherosclerosis: a potential therapeutic target for atherothrombotic vascular disease. Thromb Res 2011; 128: 117–23. doi: 10.1016/j.thromres.2011.03.025. 21636112

[7] Grote K, Luchtefeld M, Schieffer B JANUS under stress – role of JAK/STAT signaling pathway in vascular diseases. Vascul Pharmacol 2005;43:357–63. doi: 10.1016/j.vph.2005.08.021. 16271517

[8] Sun H, Wang Y Interferon regulatory factors in heart: stress response beyond inflammation. Hypertension 2014; 63: 663–4. doi: 10.1161/hypertensionaha.113.02795. 24396026 PMC4046326

[9] Nguyen H, Hiscott J, Pitha PM The growing family of interferon regulatory factors. Cytokine Growth Factor Rev 1997; 8: 293–312. doi: 10.1016/S1359-6101(97)00019-1. 9620643

[10] Andersen P, Pedersen MW, Woetmann A, Villingshoj M, Stockhausen MT, Odum N, et al. EGFR induces expression of IRF-1 via STAT1 and STAT3 activation leading to growth arrest of human cancer cells. Int J Cancer 2008; 122: 342–9. doi: 10.1002/ijc.23109. 17918184

[11] Manea A, Tanase LI, Raicu M, Simionescu M Jak/STAT signaling pathway regulates nox1 and nox4-based NADPH oxidase in human aortic smooth muscle cells. Arterioscler Thromb Vasc Biol 2010; 30: 105–12. doi: 10.1161/atvbaha.109.193896. 19834108

[12] Samavati L, Rastogi R, Du W, Huttemann M, Fite A, Franchi L STAT3 tyrosine phosphorylation is critical for interleukin 1 beta and interleukin-6 production in response to lipopolysaccharide and live bacteria. Mol Immunol 2009; 46: 1867–77. doi: 10.1016/j.molimm.2009.02.018. 19299019

[13] Wallace TC Anthocyanins in cardiovascular disease. Adv Nutr 2011; 2: 1–7. doi: 10.3945/an.110.000042. 22211184 PMC3042791

[14] Cutler BR, Petersen C, Anandh Babu PV Mechanistic insights into the vascular effects of blueberries: evidence from recent studies. Mol Nutr Food Res 2017; 61: 1600271. doi: 10.1002/mnfr.201600271. 27558887

[15] Kulling SE, Rawel HM Chokeberry (Aronia melanocarpa) – a review on the characteristic components and potential health effects. Planta Med 2008; 74: 1625–34. doi: 10.1055/s-0028-1088306. 18937167

[16] Taheri R, Connolly BA, Brand MH, Bolling BW Underutilized chokeberry (Aronia melanocarpa, Aronia arbutifolia, Aronia prunifolia) accessions are rich sources of anthocyanins, flavonoids, hydroxycinnamic acids, and proanthocyanidins. J Agric Food Chem 2013; 61: 8581–8. doi: 10.1021/jf402449q. 23941506

[17] Wu X, Beecher GR, Holden JM, Haytowitz DB, Gebhardt SE, Prior RL Concentrations of anthocyanins in common foods in the United States and estimation of normal consumption. J Agric Food Chem 2006; 54: 4069–75. doi: 10.1021/jf060300l. 16719536

[18] Chrubasik C, Li G, Chrubasik S The clinical effectiveness of chokeberry: a systematic review. Phytother Res 2010; 24: 1107–14. doi: 10.1002/ptr.3226. 20572194

[19] Bell DR, Gochenaur K Direct vasoactive and vasoprotective properties of anthocyanin-rich extracts. J Appl Physiol (1985) 2006; 100: 1164–70. doi: 10.1152/japplphysiol.00626.2005. 16339348

[20] Naruszewicz M, Laniewska I, Millo B, Dluzniewski M Combination therapy of statin with flavonoids rich extract from chokeberry fruits enhanced reduction in cardiovascular risk markers in patients after myocardial infraction (MI). Atherosclerosis 2007; 194: e179–84. doi: 10.1016/j.atherosclerosis.2006.12.032. 17320090

[21] Alsaffar H, Martino N, Garrett JP, Adam AP Interleukin-6 promotes a sustained loss of endothelial barrier function via Janus kinase-mediated STAT3 phosphorylation and de novo protein synthesis. Am J Physiol Cell Physiol 2018; 314: C589–C602. doi: 10.1152/ajpcell.00235.2017. 29351406

[22] Cohen T, Nahari D, Cerem LW, Neufeld G, Levi BZ Interleukin 6 induces the expression of vascular endothelial growth factor. J Biol Chem 1996; 271: 736–41. doi: 10.1074/jbc.271.2.736. 8557680

[23] Ikeda U, Ikeda M, Oohara T, Kano S, Yaginuma T Mitogenic action of interleukin-1 alpha on vascular smooth muscle cells mediated by PDGF. Atherosclerosis 1990; 84: 183–8. doi: 10.1016/0021-9150(90)90089-2. 2282097

[24] Nomoto A, Mutoh S, Hagihara H, Yamaguchi I Smooth muscle cell migration induced by inflammatory cell products and its inhibition by a potent calcium antagonist, nilvadipine. Atherosclerosis 1988; 72: 213–19. doi: 10.1016/0021-9150(88)90083-4. 2850808

[25] Watson C, Whittaker S, Smith N, Vora AJ, Dumonde DC, Brown KA IL-6 acts on endothelial cells to preferentially increase their adherence for lymphocytes. Clin Exp Immunol 1996; 105: 112–19. doi: 10.1046/j.1365-2249.1996.d01-717.x. 8697617 PMC2200481

[26] Sironi M, Breviario F, Proserpio P, Biondi A, Vecchi A, Van Damme J, et al. IL-1 stimulates IL-6 production in endothelial cells. J Immunol 1989; 142: 549–53. 2783442

[27] Sawada S, Chosa N, Ishisaki A, Naruishi K Enhancement of gingival inflammation induced by synergism of IL-1beta and IL-6. Biomed Res 2013; 34: 31–40. doi: 10.2220/biomedres.34.31. 23428978

[28] Chao PY, Huang YP, Hsieh WB Inhibitive effect of purple sweet potato leaf extract and its components on cell adhesion and inflammatory response in human aortic endothelial cells. Cell Adh Migr 2013; 7: 237–45. doi: 10.4161/cam.23649. 23466865 PMC3954030

[29] Krga I, Monfoulet LE, Konic-Ristic A, Mercier S, Glibetic M, Morand C, et al.: Anthocyanins and their gut metabolites reduce the adhesion of monocyte to TNFalpha-activated endothelial cells at physiologically relevant concentrations. Arch Biochem Biophys 2016; 599: 51–9. doi: 10.1016/j.abb.2016.02.006. 26873533

[30] Amin HP, Czank C, Raheem S, Zhang Q, Botting NP, Cassidy A, et al.: Anthocyanins and their physiologically relevant metabolites alter the expression of IL-6 and VCAM-1 in CD40L and oxidized LDL challenged vascular endothelial cells. Mol Nutr Food Res 2015; 59: 1095–106. doi: 10.1002/mnfr.201400803. 25787755 PMC4950056

[31] Youdim KA, McDonald J, Kalt W, Joseph JA Potential role of dietary flavonoids in reducing microvascular endothelium vulnerability to oxidative and inflammatory insults (small star, filled). J Nutr Biochem 2002; 13: 282–8. doi: 10.1016/S0955-2863(01)00221-2. 12015158

[32] Speciale A, Canali R, Chirafisi J, Saija A, Virgili F, Cimino F Cyanidin-3-O-glucoside protection against TNF-alpha-induced endothelial dysfunction: involvement of nuclear factor-kappaB signaling. J Agric Food Chem 2010; 58: 12048–54. doi: 10.1021/jf1029515. 20958056

[33] Luo X, Fang S, Xiao Y, Song F, Zou T, Wang M, et al. Cyanidin-3-glucoside suppresses TNF-alpha-induced cell proliferation through the repression of Nox activator 1 in mouse vascular smooth muscle cells: involvement of the STAT3 signaling. Mol Cell Biochem 2012; 362: 211–18. doi: 10.1007/s11010-011-1144-3. 22120492

[34] Sen CK, Bagchi D Regulation of inducible adhesion molecule expression in human endothelial cells by grape seed proanthocyanidin extract. Mol Cell Biochem 2001; 216: 1–7. doi: 10.1023/A:1011053300727. 11216853

[35] Daniela L, Alla P, Maurelli R, Elena D, Giovanna P, Vladimir K, et al. Anti-inflammatory effects of concentrated ethanol extracts of Edelweiss (Leontopodium alpinum Cass.) callus cultures towards human keratinocytes and endothelial cells. Mediators Inflamm 2012; 2012: 498373. doi: 10.1155/2012/498373. 23093820 PMC3474292

[36] Springer TA: Traffic signals for lymphocyte recirculation and leukocyte emigration: the multistep paradigm. Cell 1994; 76: 301-14. doi: 10.1016/0092-8674(94)90337-9. 7507411

[37] Cybulsky MI, Iiyama K, Li H, Zhu S, Chen M, Iiyama M, et al. A major role for VCAM-1, but not ICAM-1, in early atherosclerosis. J Clin Invest 2001; 107: 1255–62. doi: 10.1172/jci11871. 11375415 PMC209298

[38] Iademarco MF, McQuillan JJ, Rosen GD, Dean DC Characterization of the promoter for vascular cell adhesion molecule-1 (VCAM-1). J Biol Chem 1992; 267: 16323–9. 1379595

[39] Neish AS, Read MA, Thanos D, Pine R, Maniatis T, Collins T Endothelial interferon regulatory factor 1 cooperates with NF-kappa B as a transcriptional activator of vascular cell adhesion molecule 1. Mol Cell Biol 1995; 15: 2558–69. doi: 10.1128/mcb.15.5.2558. 7537851 PMC230486

[40] Lechleitner S, Gille J, Johnson DR, Petzelbauer P Interferon enhances tumor necrosis factor-induced vascular cell adhesion molecule 1 (CD106) expression in human endothelial cells by an interferon-related factor 1-dependent pathway. J Exp Med 1998; 187: 2023–30. doi: 10.1084/jem.187.12.2023. 9625761 PMC2212361

[41] Lin JH, Zhu Y, Liao HL, Kobari Y, Groszek L, Stemerman MB Induction of vascular cell adhesion molecule-1 by low-density lipoprotein. Atherosclerosis 1996; 127: 185–94. doi: 10.1016/s0021-9150(96)05951-5. 9125308

[42] Neish AS, Williams AJ, Palmer HJ, Whitley MZ, Collins T Functional analysis of the human vascular cell adhesion molecule 1 promoter. J Exp Med 1992; 176: 1583–93. doi: 10.1084/jem.176.6.1583. 1281211 PMC2119448

[43] Papi A, Johnston SL Respiratory epithelial cell expression of vascular cell adhesion molecule-1 and its up-regulation by rhinovirus infection via NF-kappaB and GATA transcription factors. J Biol Chem 1999; 274: 30041–51. doi: 10.1074/jbc.274.42.30041. 10514490

[44] Warner EF, Smith MJ, Zhang Q, Raheem KS, O’Hagan D, O’Connell MA, et al. Signatures of anthocyanin metabolites identified in humans inhibit biomarkers of vascular inflammation in human endothelial cells. Mol Nutr Food Res 2017; 61: 1700053. doi: 10.1002/mnfr.201700053. PMC5600085 28457017

